# Clinical investigation of patients with jaw deformity with comorbidities

**DOI:** 10.1186/s40902-022-00345-7

**Published:** 2022-04-06

**Authors:** Kiyohiro Kasahara, Teruhide Hoshino, Kei Sugiura, Yuki Tanimoto, Masahide Koyachi, Masae Yamamoto, Keisuke Sugahara, Masayuki Takano, Akira Katakura

**Affiliations:** 1 Department of Oral Pathobiological Science and Surgery, Tokyo Dental College, 2-9-18 Kandamisaki-cho, Chiyoda-ku, Tokyo, 101-0061 Japan; 2 Department of Oral and Maxillofacial Surgery, Tokyo Dental College, 2-9-18 Kandamisaki-cho, Chiyoda-ku, Tokyo, 101-0061 Japan

**Keywords:** Orthognathic surgeries, Comorbidities, Hemophilia, Diabetes mellitus

## Abstract

**Background:**

With improvements in the safety and stability of surgeries, the number of orthognathic surgeries is increasing. Most patients who undergo orthognathic surgeries are younger, and the number of orthognathic surgeries for patients with comorbidities is also increasing. We report a survey and clinical investigation of patients with comorbidities who underwent orthognathic surgeries at our department to improve the safety of orthognathic surgery.

**Results:**

The participants included 296 men and 712 women, with a mean age of 28 years (13–19 years, *n*=144; 20–29 years, *n*=483; 30–39 years, *n*=236; 40–49 years, *n*=102; 50–59 years, *n*=39; ≥60 years, *n*=4). In total, 347 patients underwent one-stage Le Fort type I osteotomy and sagittal split ramus osteotomy (SSRO), 243 underwent SSRO, 287 underwent plate removal, 126 underwent genioplasty and plate removal, and five underwent other surgeries. In total, 529 patients had comorbidities (52%), including allergic diseases (*n*=220, 33%), respiratory diseases (*n*=107, 16%), neurologic and psychiatric diseases (*n*=69, 10%), gynecologic diseases (*n*=28, 4%), hematologic diseases (*n*=27, 4%), cardiovascular diseases (*n*=24, 4%), digestive diseases (*n*=22, 3%), metabolic and endocrine diseases (*n*=18, 3%), spinal diseases (*n*=11, 2%), ophthalmologic diseases (*n*=11, 2%), renal and urological diseases (*n*=9, 1%), and other diseases (*n*=117, 18%). Among the patients with comorbidities, 11 with hemorrhagic diatheses (hemophilia and von Willebrand disease), arrhythmia (atrioventricular block), psychiatric disease (adjustment disorder), and metabolic disease (diabetes) required cautious perioperative management. The patient with hemophilia was managed with regular low-dose recombinant factor VIII replacement therapy, and the patient with type I diabetes mellitus was administered continuous insulin infusion and sliding-scale insulin therapy; both patients had an uneventful course.

**Conclusions:**

The study findings suggest that with the increase in orthognathic surgeries, oral and maxillofacial surgeons should adequately manage cases requiring cautious perioperative control and highlight the importance of preoperative screening. Despite the well-established safety and postoperative stability of orthognathic surgeries, oral surgeons should adopt appropriate additional preventive measures for patients with comorbidities.

## Introduction

With improvements in the safety and stability of surgeries, the number of orthognathic surgeries is increasing. Most patients who undergo orthognathic surgeries are younger, and we are beginning to encounter more orthognathic surgeries in younger patients with comorbidities [1–5]. Despite many studies on patients with jaw deformity [6–9], few have focused on patients with jaw deformity and comorbidities requiring cautious perioperative care. We report a survey and clinical investigation of patients with comorbidities who underwent consultation and orthognathic surgeries at our department to improve the safety of orthognathic surgery.

## Participants and methods

This study was approved by the Ethics Committee of Tokyo Dental College (Approval no.: 1030). Participants included 1008 patients who consulted the Department of Oral and Maxillofacial Surgery, Suidobashi Hospital at Tokyo Dental College, and underwent orthognathic-related surgery for a diagnosis of jaw deformity between January 2018 and December 2020. Information on participants’ sex, comorbidities, and operative methods was collected. A detailed survey was conducted in cases that required cautious perioperative management.

## Results

### Patient characteristics

The participants comprised 296 men and 712 women, with a mean age of 28 years (13–19 years, *n*=144; 20–29 years, *n*=483; 30–39 years, *n*=236; 40–49 years, *n*=102; 50–59 years, *n*=39; ≥60 years, *n*=4).

In total, 347 patients underwent one-stage Le Fort type I osteotomy and sagittal split ramus osteotomy (SSRO), 243 underwent SSRO, 287 underwent plate removal, 126 underwent genioplasty and plate removal, and five underwent other surgeries.

In total, 529 patients had systemic diseases (52%), including allergic diseases (*n*=220, 33%), respiratory diseases (*n*=107, 16%), neurologic and psychiatric diseases (*n*=69, 10%), gynecologic diseases (n=28, 4%), hematologic diseases (*n*=27, 4%), cardiovascular diseases (*n*=24, 4%), digestive diseases (*n*=22, 3%), metabolic and endocrine diseases (*n*=18, 3%), spinal diseases (*n*=11, 2%), ophthalmologic diseases (*n*=11, 2%), renal and urological diseases (*n*=9, 1%), and other diseases (*n*=117, 18%).

Among the patients with comorbidities, 11 who required cautious perioperative management were transferred to and treated at the Department of Dentistry and Oral Surgery, Tokyo Dental College Ichikawa General Hospital (Table 1). Comorbidities, such as hemorrhagic diatheses (hemophilia, von Willebrand disease), arrhythmia (second-degree atrioventricular block), psychiatric disease (adjustment disorder), and metabolic disease (type I diabetes mellitus), were observed. Among these patients, two cases, one with hemophilia (case 1) and one with type I diabetes (case 10), are discussed below in detail.

**Table 1:** Patients who required cautious perioperative management

Case no.	Sex	Age (years)	Diagnosis	Comorbidity	Medical history	Operative method	Operative time (min)	Blood loss (mL)
1	Male	27	Anterior cross-bite with mandibular prognathism	Hemophilia A	Pulmonary stenosis, pediatric asthma	SSRO	140	150
2	Female	29	Anterior cross-bite with maxillary retrognathism, mandibular prognathism)	Arrythmia (second-degree Wenckebach AV block)	None	Le Fort I SSRO	250	100
3	Female	33	Open-bite with maxillary prognathism and mandibular retrognathism	Liver disorder	None	Le Fort I SSRO	271	150
4	Female	30	Open-bite with maxillary prognathism and mandibular retrognathism	Arrythmia (second-degree Wenckebach AV block)	None	Le Fort I SSRO	230	Small amount
5	Female	34	Anterior cross-bite with mandibular prognathism	von Willebrand disease	Obsessive-compulsive disorder	SSRO	137	Small amount
6	Female	49	Anterior cross-bite with mandibular prognathism	Epilepsy	None	SSRO	182	50
7	Male	56	Anterior cross-bite with mandibular prognathism	Arrhythmia (atrial fibrillation, left contractile dysfunction)	Chronic heart failure	SSRO	160	50
8	Female	25	Anterior cross-bite with maxillary prognathism and mandibular retrognathism	Psychiatric disorder (adjustment disorder)	None	Plate removal	113	Small amount
9	Male	36	Anterior cross-bite with mandibular prognathism	Labyrinthe disorder (vertigo, auditory disturbance), low-frequency allergy	None	SSRO	155	50
10	Female	32	Anterior cross-bite with mandibular prognathism	Type I diabetes mellitus	None	Le Fort I SSRO	300	20
11	Male	19	Anterior cross-bite with facial asymmetry and mandibular prognathism	Epilepsy	None	SSRO	151	Small amount

*SSRO* sagittal split ramus osteotomy, *AV* atrioventricular

### Case 1

The patient was a 20-year-old man with a chief complaint of an underbite. He had a medical history of asthma and pulmonary valve stenosis. He became aware of his mandibular prognathism when he was in his first year of junior high school. Although he started orthodontic treatment in the first year of senior high school, the mandibular prognathism did not improve. The patient desired to undergo surgical orthodontic therapy and consulted our hospital in 2014. The extraoral findings were mandibular deviation to the right on the frontal view and severe mandibular prognathism with a concave profile on the profile view. Furthermore, the patient experienced temporomandibular joint symptoms of clicking when opening his mouth. The intraoral findings were bilateral angle class III malocclusion, overbite of 0 mm, and overjet of − 2 mm. The clinical diagnosis was anterior cross-bite with jaw deformity (mandibular prognathism). The cephalometric radiogram, which was evaluated by an orthodontist, revealed an anterior cross-bite with mandibular prognathism requiring bilateral SSRO for improvement. No abnormal findings were observed in a general screening test before initiating the preoperative orthodontic treatments. However, a coagulation system test was not performed, and the patient was not screened for hemorrhagic diatheses. The patient visited our department again in April 2018, when the preoperative orthodontic treatment was completed.

**Fig. 1:**
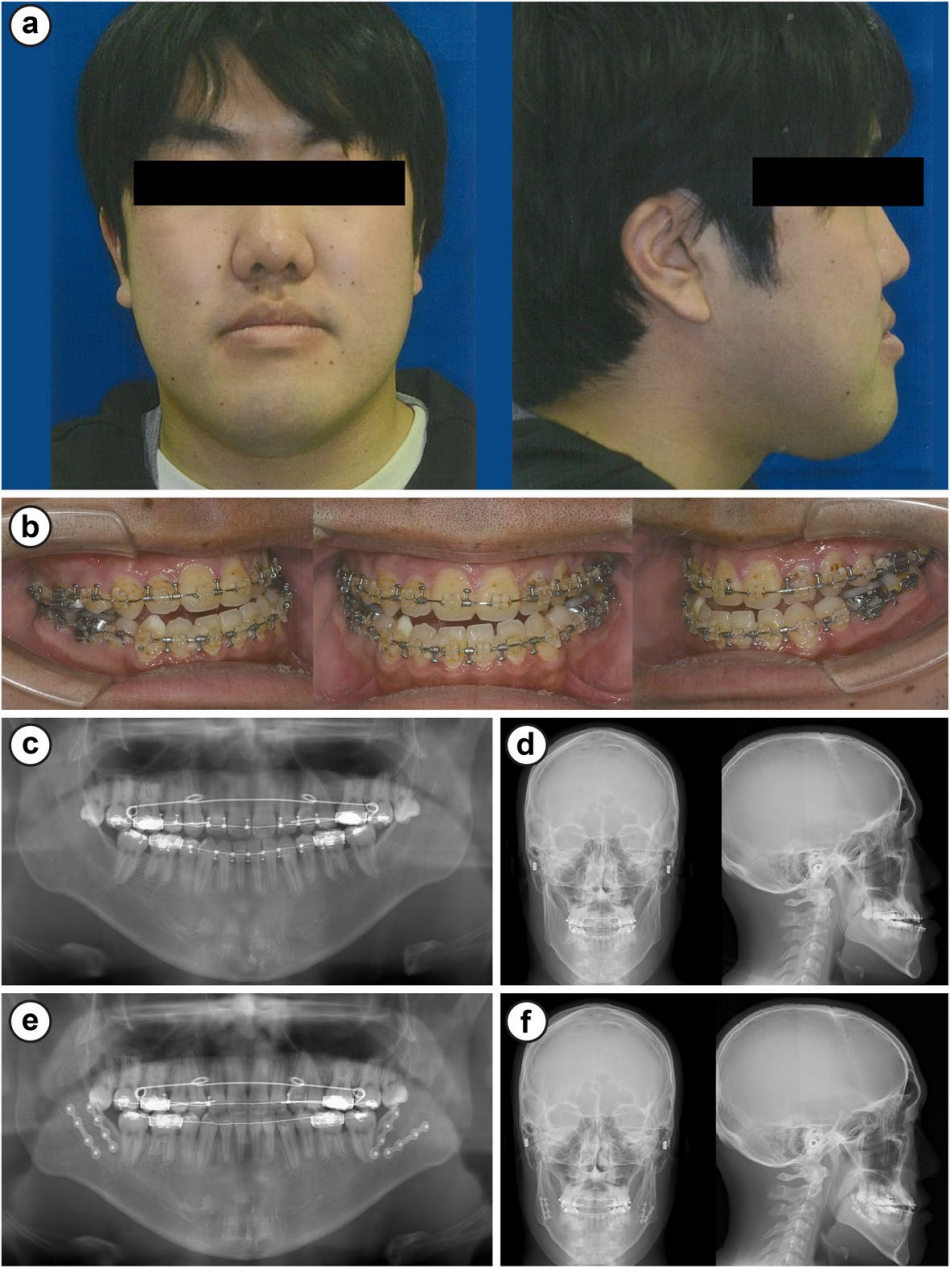
Case 1. **a** Facial photographs, **b** intraoral images, **c** panoramic radiographs, **d** cephalometric radiogram, **e** panoramic radiographs 1 month postoperatively, and **f** cephalometric radiogram 1 month postoperatively

Preoperatively, the extraoral findings included severe mandibular prognathism and a concave profile on profile view (Fig. 1a), and the intraoral findings included bilateral angle class III malocclusion, overbite of 0 mm, and overjet of − 8 mm (Fig. 1b). Panoramic radiographs revealed that the lower wisdom teeth and upper and lower first premolars were bilaterally extracted; however, the upper wisdom teeth were present (Fig. 1c, d). The cephalometric radiogram revealed the following findings: sella/nasion plane and nasion/A plane angle (SNA), 74.2°; sella/nasion plane and nasion/B plane angle (SNB), 80.2°; and A point–nasion–B point (ANB), −6°. Wits analysis revealed −14.8 relative mandibular prognathism (Fig. 1d) (Table 2). The clinical diagnosis was anterior cross-bite with jaw deformity (mandibular prognathism).

**Table 2:** Preoperative analysis results: case 1

Measurements (degrees)	Patient	Adult male
	Initial	Mean±standard deviation
Facial angle	88.8	85.9±1.7
FMA	37.3	26.4±3.8
Y-axis	67.7	64.3±2.3
Gonial angle	130.7	120.2±4
SNA	74.2	83.2±3.4
SNB	80.2	80.4±3.2
ANB	−6	2.8±1.8
Occlusal plane	7.5	8.6±5.6
Interincisal angle	131.1	121.9±3.7
L1 to mandibular	73.8	98.2±4.1
U1 to the FH plane	117.8	115.2±5.9
Wits appraisal	−14.8	1.1±1.9

*FMA* Frankfort–mandibular plane angle, *SNA* sella/nasion plane and nasion/A plane angle, *SNB* sella/nasion plane and nasion/B plane angle, *ANB* A point–nasion–B point, *FH* Frankfort horizontal plane

**Fig. 2:**
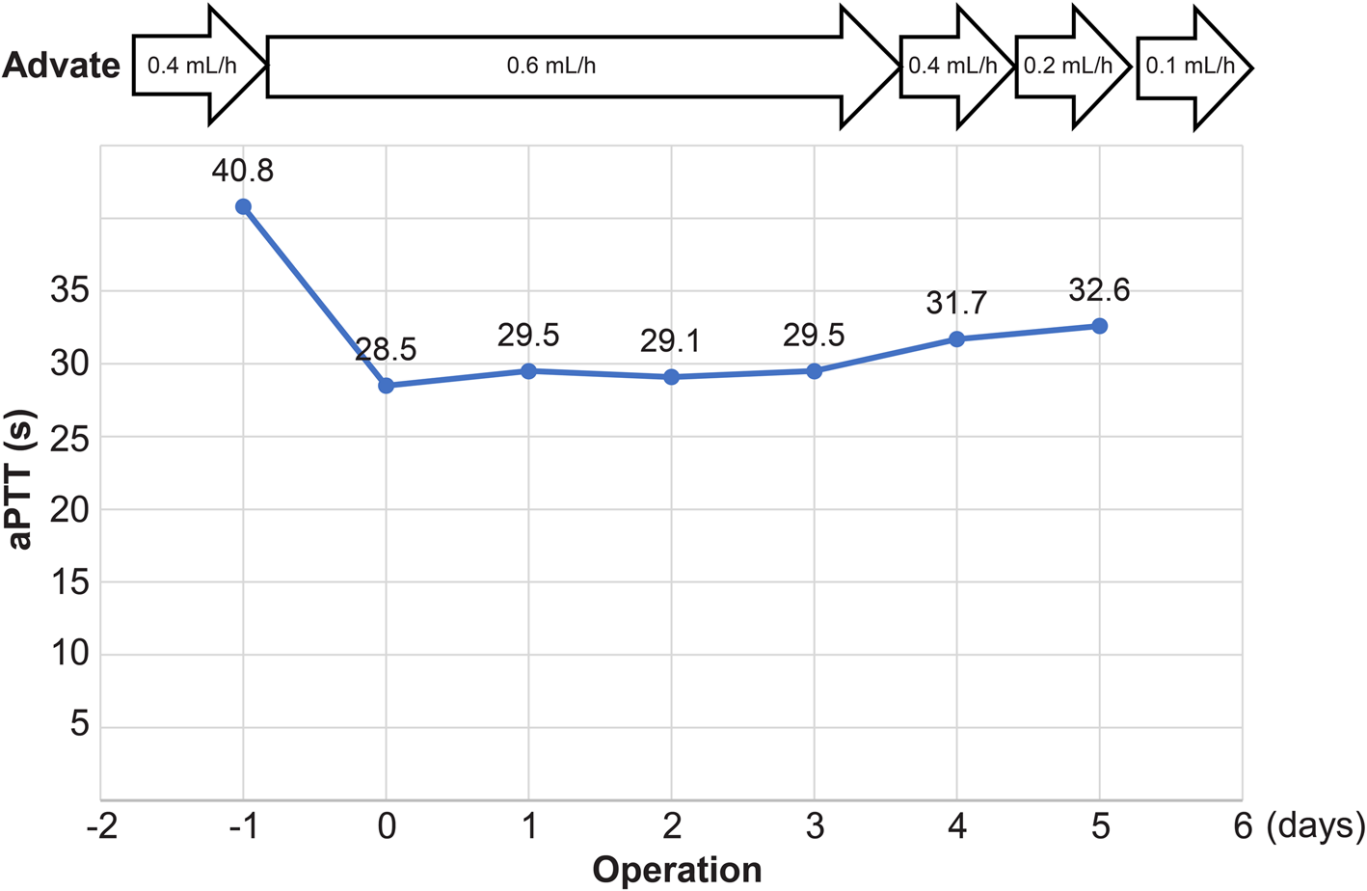
Changes in activated partial thromboplastin time (aPTT)

As with the first examination, bilateral SSRO was planned based on the results of the analysis of the cephalometric radiograms taken in September 2019. In a screening test immediately before the surgery, a prolonged activated partial thromboplastin time (aPTT, 47.9 s) was noted. Detailed examinations performed at the Department of Internal Medicine and Hematology at another facility showed low factor VIII activity (11%), and the patient was diagnosed with mild hemophilia A. Before surgery, the patient was informed about the precautionary measures required for preoperative and postoperative bleeding, and he opted to push through with the surgery. He was transferred to the Department of Dentistry and Oral Surgery, Tokyo Dental College Ichikawa General Hospital, and together with our hematology department, surgery was planned. Surgical invasiveness and expected blood loss were considered; thus, autologous blood was collected, and recombinant factor VIII replacement therapy (Advate; Takeda, Tokyo, Japan) was administered preoperatively. Low-dose replacement therapy was administered to avoid factor VIII inhibitors induced by initial high-dose therapy. Immediately before surgery, the aPTT was 40.8 s, factor VIII activity was 21%, and the patient was negative for factor VIII inhibitors. Bilateral SSRO was performed under general anesthesia (Fig. 4e, f). The 140-min surgery was completed without abnormal intraoperative bleeding with a blood loss of 150 mL. Recombinant factor VIII replacement therapy (Advate) was continuously administered postoperatively. The aPTT level was stable; thus, Advate was tapered and discontinued on day 8 (Fig. 2). The continuous suction drain was removed 2 days later. No abnormal bleeding was observed during the hospital stay, and the patient was discharged. The postoperative course at 1 year was good (Fig. 1e, f).

### Case 10

The patient was a 30-year-old woman with a chief complaint of an underbite. She had a history of anterior cross-bite since primary dentition. Mandibular prognathism was observed by an orthodontist at approximately 20 years old; however, the patient stopped visiting the clinician. The patient visited the department again in February 2019 and was recommended for surgical orthodontic therapy at the Department of Orthodontics at our hospital, and she was transferred to our department. She had type I diabetes, pediatric asthma, and drug allergy (propionic acid derivative non-steroidal anti-inflammatory drug). The extraoral findings were severe mandibular prognathism and a concave profile on profile view. Conversely, the intraoral findings were bilateral angle class III malocclusion, a mandibular deviation to the left, overbite of 0 mm, overjet of −6 mm, and the right maxillary second premolar and left mandibular central incisor were defective. Lingual tipping of the mandibular anterior teeth was also observed. The clinical diagnosis at initial examination was anterior cross-bite with jaw deformity (mandibular prognathism).

Analysis of the cephalometric radiogram by the orthodontist at her first examination at our department showed anterior cross-bite with maxillary retrognathism and mandibular prognathism requiring Le Fort type I osteotomy with bilateral SSRO for improvement. The patient’s endocrinologist managed the patient’s type I diabetes mellitus with insulin lispro (8-8-8-0 units, Humalog Mix Injection; Eli Lilly, Carolina, Puerto Rico) and insulin detemir (0-0-0-10 units, Levemir FlexPen; Novo Nordisk, Clayton, NC, USA), and the patient achieved good glycemic control, with stable glycated hemoglobin level (HbA1c) between 5 and 6%. The patient was informed that surgery was feasible under strict systemic management, and she opted for surgery. In the general screening before starting preoperative orthodontic treatment, the patient’s fasting blood glucose and HbA1c were 108 mg/dL and 5.8%, respectively. The course was good after the lower left wisdom tooth and upper left first premolar extraction, and there were no signs of susceptibility to infection or delayed wound healing. The patient visited our department again in January 2020, when the preoperative orthodontic treatment was completed.

**Fig. 3:**
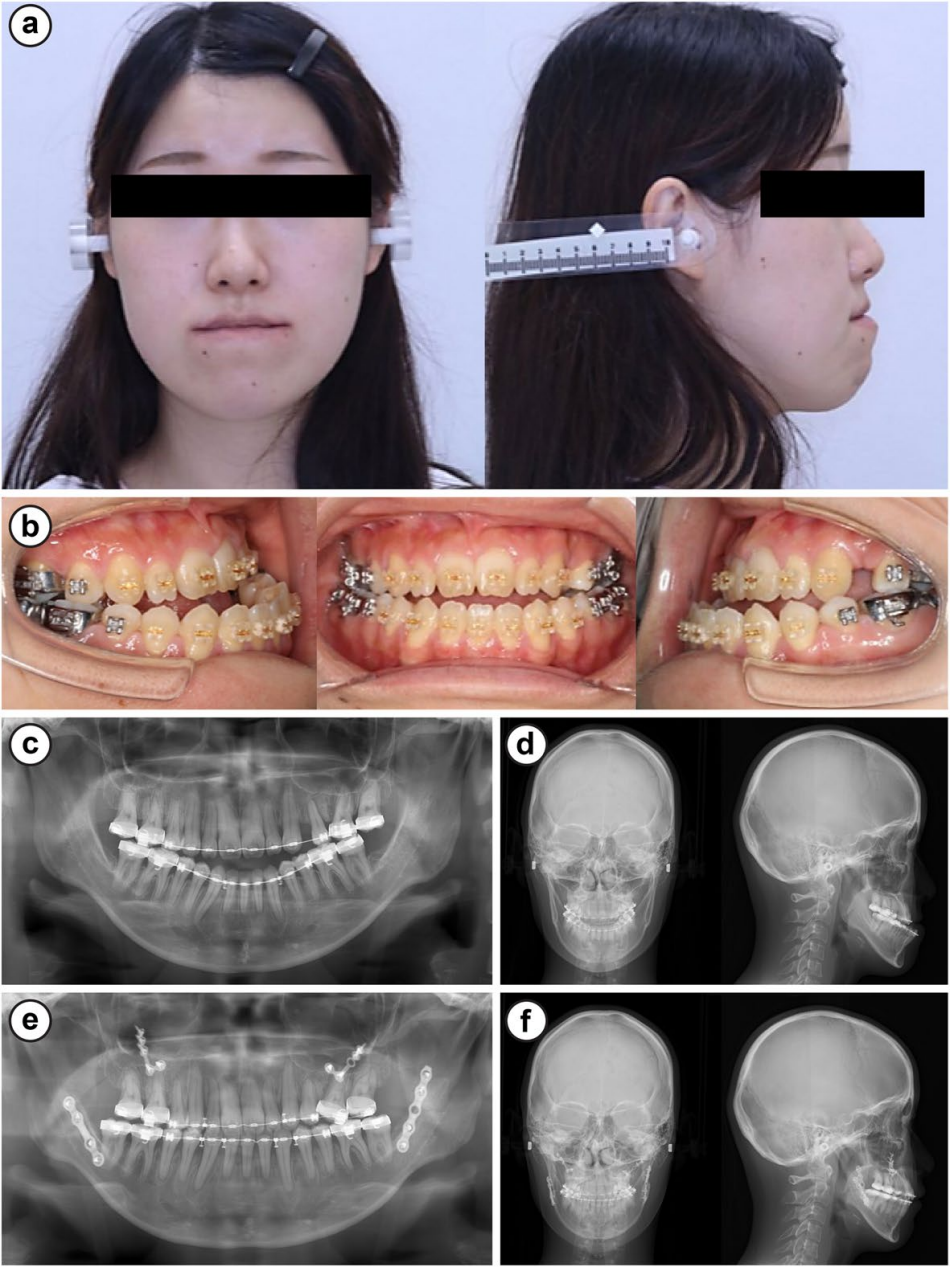
Case 10. **a** Facial photographs, **b** intraoral images, **c** panoramic radiographs, **d** cephalometric radiogram, **e** panoramic radiographs 1 year 6 months postoperatively, and **f** cephalometric radiogram 1 year 6 months postoperatively

Preoperatively, the extraoral findings were severe mandibular prognathism and a concave profile in profile view (Fig. 3a), whereas the intraoral findings were bilateral angle class III malocclusion, overbite of 0 mm, and overjet of −8 mm (Fig. 3b). Panoramic radiographs revealed that the upper and lower wisdom teeth and upper left first premolars were extracted (Fig. 3c). The cephalometric radiogram revealed the following findings: SNA, 81.2°; SNB, 83.6°; and ANB, −2.4°. Wits analysis revealed −19.5 mandibular prognathism (Fig. 3d) (Table 3). Therefore, the clinical diagnosis was anterior cross-bite with jaw deformity (maxillary retrognathism and mandibular prognathism).

**Table 3:** Preoperative analysis results: case 10

Measurements (degrees)	Patient	Adult male
	Initial	Mean±standard deviation
Facial angle	88.2	87.8±3.6
FMA	34.2	25±6
*Y*-axis	65.4	59.4±3.6
Gonial angle	136	120.2±4
SNA	81.2	82±3
SNB	83.6	80±3
ANB	−2.4	2±2
Occlusal plane to SN	25.3	9.3±3.8
Interincisal angle	133.5	124±6
L1 to mandibular	86.4	90±6
Wits appraisal	−19.5	1.1±1.9

*FMA* Frankfort–mandibular plane angle, *SNA* sella/nasion plane and nasion/A plane angle, *SNB* sella/nasion plane and nasion/B plane angle, *ANB* A point–nasion–B point

**Fig. 4:**
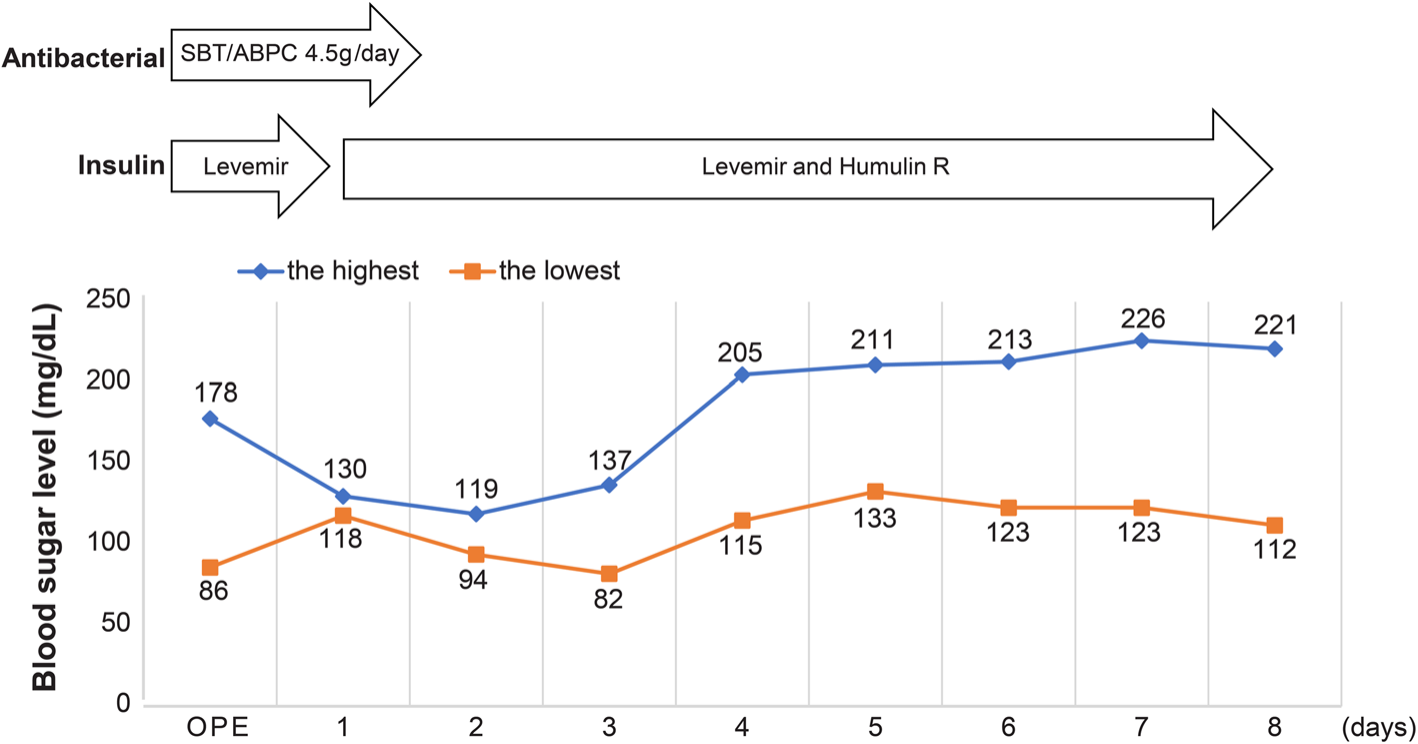
Changes in glycemic levels

As with the first examination, Le Fort type I osteotomy with bilateral SSRO was planned according to the results of the cephalometric radiogram taken in August 2020. In the preoperative screening test, the patient’s fasting glycemic level and HbA1c were 166 mg/dL and 6.5%, respectively. A second consultation with the patient’s diabetologist revealed that the patient was on insulin lispro (6-6-6-0 units, Humalog Mix Injection; Eli Lilly), insulin detemir (0-0-0-6 units, Levemir FlexPen; Novo Nordisk), and oral dapagliflozin propylene glycolate hydrate (5 mg, Forxiga; AstraZeneca, Osaka, Japan) for glycemic control. HbA1c was approximately 6%, suggesting a relatively stable condition without hypoglycemia. The potential complications, such as susceptibility to infection and delayed wound healing, were explained to the patient before the surgery. Le Fort type I osteotomy with bilateral SSRO was performed under general anesthesia. Moreover, insulin (human recombinant; Humulin R; Eli Lilly) was administered intravenously during the surgery according to the instructions of the patient’s diabetologist, with hourly testing of blood glucose levels to adjust the dose accordingly. The patient did not experience hypoglycemia. Postoperatively, the oral diet was expected to be unstable given the invasiveness of the surgery and intermaxillary fixation; hence, the blood glucose levels were measured four times a day and managed on a sliding scale with insulin lispro (Humalog Mix Injection; Eli Lilly) and insulin detemir (Levemir FlexPen; Novo Nordisk). Since glycemic control with insulin was good, dapagliflozin propylene glycolate hydrate (Forxiga tablets) was discontinued. The patient had a good postoperative course without hypoglycemia (Fig. 4) and was discharged without postoperative wound infection or delayed wound healing. The postoperative course at 1 year was uneventful (Fig. 3e, f).

## Discussion

Our hospital is located in the Tokyo metropolitan area and is affiliated with a dental college. Approximately 8000 new patients visited the Department of Oral and Maxillofacial Surgery in 2013, of whom 301 (0.03%) had jaw deformities [10]. However, as orthognathic surgery has become more widely known, the number of orthognathic surgeries being performed is increasing [11]. Our hospital is one of Japan’s facilities with the highest volumes of orthognathic surgeries, and in recent years, the safety, postoperative stability, and reduction of blood loss and operative times of orthognathic surgeries are increasingly improving [11]. Patients requiring perioperative care because of bleeding tendency and those with deterioration of general state or restlessness postoperatively are transferred to Tokyo Dental College Ichikawa General Hospital. Transfers are determined according to preoperative screening results and/or the presence of any comorbidities. In terms of preoperative screening, blood tests and electrocardiograms (ECGs) are performed twice, once before preoperative orthodontic treatment and again immediately before the surgery, during which the results are analyzed and considered by an internist and a dental anesthesiologist. Patients with a systemic condition that prevents safe operation under general anesthesia as per the screening test before the preoperative orthodontic treatment are treated for the primary disease before initiating orthodontic treatment.

Many patients with jaw deformity who consult orthognathic departments for surgical treatment to achieve normal occlusion also hope to improve facial appearance, which may explain the predominance of female patients [12]. The same trend in the sex ratio was observed among patients who consulted our department. Most of our patients were in their 20s because this age period coincides with the end of the jaw and facial bone development, which is when orthognathic surgery is normally performed, as this age group is just about to join the workforce, and the majority of new patients in our department are in their 20s and 30s [10]. Recently, two-jaw surgeries are being increasingly performed to improve facial appearance [13], and the higher number of two-jaw surgeries at our department reflected this trend. In our department, one-stage genioplasty with two- or one-jaw surgery is avoided to prevent injury to the inferior alveolar and mental nerves, and geniosplasty is performed simultaneously with plate removal. Several studies have reported on orthognathic surgeries performed in patients with comorbidities [1–5]. However, the number of congenital and acquired diseases is low. More than half (52%) of the patients in the present study had comorbidities, among which 11 were determined to require cautious perioperative management. Since the majority of patients in their 20s and 30s have never undergone general anesthesia and many have never undergone a blood test or ECG at a hospital, preoperative testing is extremely important to screen for any problems preoperatively. At times, congenital or acquired diseases that even the patient was unaware of are discovered in this screening process. The patient with hemophilia A in this study had a previous tooth extraction under local anesthesia; however, no abnormal postoperative bleeding was observed at the time. The patient had never been diagnosed with hemophilia until it was identified by preoperative screening for orthognathic surgery. As the number of cases increases, cases such as this should be considered.

Since the patient with hemophilia A had not undergone coagulation system testing in the screening tests before preoperative orthodontic treatment, the prolonged aPTT was only discovered immediately before the surgery. This likely occurred because the attending physician decided to omit detailed examinations based on the patient’s age and absence of relevant medical history. This case highlights the importance of exhaustive testing for all potential risks of surgery, including the coagulation system, in the screening before preoperative orthodontic treatment as well as cautious monitoring and preparing for additional measures for any other problems related to diseases that the patient might have developed in the 1- to 2-year period that preoperative orthodontic treatment typically takes to ensure safe surgery under general anesthesia. This is especially true for middle-aged and older patients who are more likely to develop new comorbidities than younger patients. Therefore, it is extremely important to explain this thoroughly to the patient before starting preoperative orthodontic treatment and to encourage the patient to notify the physician in case this happens; hence, it is equally important to establish trust in the relationship with the patient before reaching that stage. The patient with hemophilia A in this study was managed with several strategies planned preoperatively, including shortening the operative time to minimize bleeding, careful hemostasis by applying local hemostatic agents, and collecting autologous blood for transfusion. The operative time was not significantly prolonged, and the blood loss was not significantly increased compared with those of other institutions [14], indicating the effectiveness of the preventive measures. Furthermore, autologous blood transfusion was not necessary. Regular low-dose recombinant factor VIII replacement therapy (Advate) was initiated preoperatively by working closely with the hematologist, allowing the aPTT and factor VIII activity to improve immediately before surgery and prevent factor VIII inhibitors, thus enabling safe surgery. When the surgery was planned, the patient was scheduled for bilateral SSRO only. Although patients with maxillary retrognathism or facial asymmetry may also require maxillary osteoplasty, it is difficult to create a closed environment with the maxilla, which can be an obstacle to achieving postoperative compression hemostasis. For patients with hemorrhagic diatheses, treatment strategies should be decided through careful discussion with an orthodontist, even if maxillary repositioning is necessary to achieve occlusion.

Type I diabetes, also known as insulin-dependent diabetes, although less frequent than type II diabetes, is common among younger people [15]. Furthermore, glycemic control is more difficult to achieve in type I than in type II diabetes, thus requiring more stringent management. Often, patients are not aware that they have type I diabetes, and among young patients, they are often discovered incidentally when they are tested for another purpose. Since orthognathic surgeries are elective surgeries, strict management can be planned preoperatively for patients with poorly controlled diabetes. The risk of abnormal fractures has been reported to increase in patients with diabetes [16]. Even with careful and protective surgical maneuvers and osteosynthesis, excessive load on the bone around the screws should be avoided. We considered using resorptive plates for osteosynthesis in this patient; however, with extensive repositioning of 10 mm on the left side and considering the absence of any problems on the cortical bone thickness, osteosynthesis was performed with titanic miniplates. In addition to a potential postoperative increase in glycemic levels due to inflammation, stress, and steroid therapy for surgical invasiveness, delayed wound healing and susceptibility to infection as well as poor glycemic control associated with decreased food intake were possible. The risk of complications reportedly decreases significantly by maintaining postoperative glycemic levels ≤180 mg/dL [17]. In this study, the patient’s glycemic levels were well controlled at ≤180 mg/dL intraoperatively and on postoperative days 0 and 1. Dietary intake, which was a point of concern, did not decrease notably, and no special adjustments were made to the duration or methods of intermaxillary fixation. However, to introduce oral intake in the early postoperative period, the wound and general oral conditions were checked once a day in an examination room by a dentist, followed by a check on patients brushing in their rooms. Furthermore, patients are taught oral hygiene by a dental hygienist before surgery to implement measures in case of wound infection.

The two main objectives of preoperative tests are to screen for any diseases that could be predicted from information obtained by the anamnesis and to obtain information that could not be determined from it. The latter is particularly important for patients undergoing orthognathic surgery because the majority of such patients are young. Hematological tests, ECG, and chest radiography are performed in our hospital during preoperative screening. A previous study reported that it is unusual to observe data in an ECG or chest cardiography of young patients that would require modification of the surgical or anesthetic plan and that patient age or information obtained from an anamnesis is more important [18]. However, the patient had no subjective symptoms of the disease that was discovered only in preoperative screening, and this emphasizes the utmost importance of performing preoperative screening in patients without exceptions before starting preoperative orthodontic treatment.

In the future, orthognathic surgeries for patients with various systemic diseases and other backgrounds are expected to increase. As orthognathic surgery is an elective procedure, whether it is necessary for the patient in the first place should also be discussed carefully with an orthodontist. Furthermore, to ensure patient satisfaction with the treatment, it is imperative to explain the surgery to the patient so that the postoperative outcome can be visualized. The safety of the operative methods and postoperative stability are increasingly being established; however, ensuring appropriate measures for cases that require cautious perioperative management remains the most important key in performing orthognathic surgeries for patients with comorbidities.

## Conclusions

The findings of this study suggest that oral and maxillofacial surgeons should adequately manage cases requiring cautious perioperative control during orthognathic surgeries and highlight the importance of preoperative screening.

## Data Availability

The data that support the findings of this study are available from the corresponding author upon reasonable request.

## Abbreviations

ANB: A point–nasion–B point
aPTT: Activated partial thromboplastin time
ECG: Electrocardiogram
FMA: Frankfort–mandibular plane angle
HbA1c: Glycated hemoglobin level
SNA: Sella/nasion plane and nasion/A plane angle
SNA: Sella/nasion plane and nasion/A plane angle
SSRO: Sagittal split ramus osteotomy
